# Genome-Wide Identification of MIKCc-Type MADS-Box Family Gene and Floral Organ Transcriptome Characterization in Ma Bamboo (*Dendrocalamus latiflorus* Munro)

**DOI:** 10.3390/genes14010078

**Published:** 2022-12-27

**Authors:** Deming Yang, Jing Yang, Jiayi Wan, Yanping Xu, Lei Li, Jundong Rong, Lingyan Chen, Tianyou He, Yushan Zheng

**Affiliations:** 1 College of Forestry, Fujian Agriculture and Forestry University, Fuzhou 350002, China; 2 College of Landscape Architecture, Fujian Agriculture and Forestry University, Fuzhou 350002, China

**Keywords:** bamboo, floral organ, MADS-box, *Dendrocalamus latiflorus* Munro, pollen abortion

## Abstract

Most bamboos die after flowering, and the molecular mechanisms responsible for flowering is poorly understood. The MIKCc-type MADS-box family gene is involved in the flowering process. To explore the mechanism of the MIKCc-type MADS-box gene and phytohormone regulation in the flowering of *Dendrocalamus latiflorus* Munro (*D. latiflorus*), characterized by extremely rapid growth and widely cultivated woody bamboo, we initially did a genome-wide analysis of the MIKCc-type MADS-box gene in *D. latiflorus*. In the meantime, transcriptome analysis was performed using the floral organs. A total of 170 MIKCc-Type MADS-Box genes were identified and divided into 15 categories. The *cis*-acting element analysis in promoters regions revealed that MIKC-type MADS-box family genes were associated with hormones, including auxin, abscisic acid (ABA), gibberellin (GA) and jasmonic acid (JA), which was found at 79, 476, 96, 486 sites and cover 61, 103, 73, 128 genes. Genome synteny analysis showed subgenome AA and BB were better than CC and obtained 49, 40, 39 synteny genes compared with *Oryza sativa* (*O. sativa*). In transcriptome analysis of floral organs, the enriched pathway from DEGs included circadian, vernalization and gibberellin pathways associated with the flowering process. We found that the jasmonic acid synthesis gene is highly expressed in the pistil, which may be the cause of Ma bamboo pollen abortion. The expression profile showed that most MIKC-type MADS-box genes exhibited high expression in flower organs. The consequences of this study will provide insight into the irregular flowering and low pollen counts of Ma bamboo.

## 1. Introduction

Bamboo shows great diversity, with more than 2000 species. They have similar phenotypes but differ in their flowering behavior, including species that bloom annually and species that flower irregularly (3–120 years or more) [1,2]. Most bamboos die after flowering [3], suggesting that a special genetic mechanism controls the floral transition. Another distinguishing feature of woody bamboo is that a stand often flowers synchronously [4], disrupting both the supply chain within the bamboo industry and affecting the local ecology. Until now, the systematic mechanism of bamboo flowering has not been clearly understood. Most research has focused on discussing flowering behavior and identifying the role of certain genes [5,6,7]. However, studies on the development of bamboo flower organs have not been reported.

Biochemical pathways of flowering include vernalization, photoperiod, circadian clock, gibberellin pathway, temperature and age [8]. How they regulate bamboo flowering is not clear. *D. latiflorus* is a woody clumping bamboo that flowers irregularly. It is widely cultivated in tropical and subtropical regions and has high economic value [5,9]. The genome sequencing of *D. latiflorus* has been reported [10], and a gene editing system [11] has been constructed. This high-quality hexaploid genome may pave the way for bamboo research using *D. latiflorus* as a model species, but the inability to control flowering is a restriction. Not only that, low amounts of pollen and seeds also suggest a special mechanism in the *D. latiflorus* that should be figured out.

MADS-box proteins are essential transcription factors for plant growth and are involved in virtually all aspects of plant development, including roots, flowers, seeds, and embryos [12,13]. They can be divided into two distinct phylogenetic groups: Type I and Type II. The type II genes are also known as MIKCc-type MADS-box genes because of the domain structure of this trait [14]. Previous studies have already shown that the MIKCc-type MADS-box gene plays an crucial role in the ABCDE model [15], involved in flowering regulation. Therefore, understanding how the MIKCc-type MADS-box gene controls the bamboo gene is of great significance for understanding flower organ development and bamboo flowering. Incidentally, the hormones produced are thought to induce bamboo flowering under drought stress [16]; gibberellin metabolism is one of the most important pathway of flowering [17,18]; in in vitro experiments, bamboo flowering was also induced by the addition of hormones to the culture medium [19]. This proves that hormones are key chemicals in the flowering of bamboo. However, the regulatory mechanism of MIKCc-type MADS-box family genes and hormones in bamboo remains unclear.

Both *O. sativa* (flower regularly) and *D. latiflorus* (flowers irregularly) belong to the Poaceae. As a model plant, the MIKCc-type MADS-box gene has been extensively studied in rice [20,21]. It was found that *OsMADS1*, which is one of the MADS-box genes of rice mutation affecting rice flowers, causes a decrease in stamen number [22]. After the initiation of floral organ primordia, the expression of *OsMADS14* [23] is restricted to sterile glumes, palaces and lemmas. Interestingly, in mature spikelets, *OsMADS14* switches to the reproductive organs, the stamens and carpels, and its expression in the sterile organs is no longer observed. These studies make the comparison between rice and Ma bamboo interesting. Transcriptome sequencing has been applied to the nutritive organ [24], and some flowering studies have also identifed several metabolic pathways in bamboo [5]. However, the transcriptome sequence of floral organs has not been determined.

In order to study the mechanism of the MIKCc-type MADS-box gene and phytohormone regulation in the flowering of *D. latiflorus*. We identified and analyzed the MIKCc-type MADS-box gene family, combined with transcriptome sequence of flower organs and synteny analysis with rice, a grain model plant, to provide further evidence for bamboo flowering.

## 2. Materials and Methods

### 2.1. Plant Materials, cDNA Synthesis and Transcriptome Sequencing

In general, the floral organ tissues including stamen, pistil and palea were collected from a bamboo farm of *D. latiflorus* in Zhangzhou, Fujian province of China (117°250′ E 24°280′ N) in August 2022. The collected flower tissues of *D. latiflorus* were immediately frozen in liquid nitrogen, and the flower organs were separated by ice for RNA extraction. Each tissue sample contained three biological replicates, and each replica was composed of tissues from three individuals. A Plant RNA Isolation Kit (Omega, CA, USA, Cat. No. R6827-02) was used to extract total RNA. The integrity of RNA samples was evaluated using an Agilent 2100 Bioanalyzer (Agilent Technologies, Santa Clara, CA, USA), and samples with RIN values higher than 8 were used for downstream RNA-Seq library construction using the dUTP method with the Illumina NovaSeq platform as 150-nt paired-end [25] reads. It was reverse-transcribed to cDNA using a PrimeScript™ RT reagent kit with gDNA Eraser (TaKaRa, Kusatsu City, Japan, Cat. No. RR047A), according to the instructions of the manufacturer. Then the cDNAs were diluted at 1:10 with RNase-free water and stored at −20 °C for qRT-PCR analyses.

### 2.2. RNA-seq Bioinformatics Analysis

The clean RNA-seq reads were mapped to the *D. latiflorus* genome using HiSAT2 software [26] with default parameters, and fragments per kilobase of transcript per million mapped (FPKM) reads were calculated using the StringTie program [26] with default parameters. Then the fold change of FPKM expression values > 2 and FDR < 0.01 were considered to be the threshold for the identification of the differentially expressed genes. The *p*-value was calculated by the statistical package EdgeR [27]. GO enrichment analysis was performed using Clusterprofiler software [28].

### 2.3. Sequence Search and Identification of MIKCc-Type MADS-Box Genes

The reference genome and annotation file of *D. latiflorus* were retrieved from the NCBI (https://www.ncbi.nlm.nih.gov, accessed on 8 September 2022) under accession PRJNA600661. *Arabidopsis* MIKCc-type MADS-box gene sequences were obtained from the TAIR database (http://www.arabidopsis.org, accessed on 8 September 2022), as queries used local alignment search tools (BLASTP) against the *D. latiflorus*. Functional annotations were filtered for Protein Family database (Pfam, https://www.ebi.ac.uk/Tools/pfa/pfamscan/, accessed on 8 September 2022) identifiers of the MADS and K domains (PF00319 and PF01486) using the HMMER (Version 3.3.2) [29] with default parameters. The obtained sequences were filtered by the following two criteria: (Step 1) sequences were removed if the sequence lengths were less than the length of the conserved motif (33 bp); (Step 2) removed sequences with gap (‘N’) > 10 bp for additional analysis.

### 2.4. Phylogenetic Tree Construction

To construct a MIKCc-type MADS-box gene phylogeny tree using MEGA (Version 6.06) [30], aligned protein sequences from three plant species, *Arabidopsis*, *O. Sativa*, and *D. latiflorus* were employed. The phylogenetic tree was constructed by applying the neighbor-joining method with amino acid p-distance, and the reliability was obtained by bootstrapping 1000 times.

### 2.5. Genome Synteny and Gene Synteny Analysis

*D. latiflorus* data from NCBI (https://www.ncbi.nlm.nih.gov, accessed on 11 September 2022) under accession PRJNA600661. Rice data from EmsembIPlant (https://plants.ensembl.org/, accessed on 11 September 2022). A multiple collinearity scan toolkit (MCScanX) [31] was adopted to identify the synteny relationship of homologous MIKCc-type MADS-box genes obtained from *D. latiflorus* and *O. Sativa*.

### 2.6. Prediction of Cis-Regulatory Elements of Promoter Region

The 1500 bp upstream sequences of *D. latiflorus* MIKCc-type MADS-box genes were extracted from whole-genome sequence and were analyzed using PlantCARE [32] promoter analysis tool (http://bioinformatics.psb.ugent.be/webtools/plantcare/html/, accessed on 15 September 2022) with default parameters for the prediction of various *cis*-acting regulatory elements. Hormone-related *cis*-acting elements were selected for further analysis.

### 2.7. RT-qPCR Validation

We used proprietary software with Quant Studio 6 (Life Technologies, Carlsbad, CA, USA) and GoTaq^®^ qPCR Master Mix (PROMEGA, Madison, WI, USA, Cat. No. A6002) for RT-qPCR reactions (Table S1). The reaction system was as follows: 2 × MasterMix 10 µL, Primer F 1 µL (10 µM), Primer R 1 µL (10 µM), cDNA 1 µL, and nuclease-free water up to 20 µL. The reaction procedure was as follows: 95 °C for 30 s; 95 °C for 15 s, 60 °C for 30 s, 72 °C for 1 min/Kb, 40 cycles. The melt curve was analyzed immediately after the reaction was completed, and the procedure was as follows: 95 °C for 15 s; 60 °C for 1 min; 95 °C for 15 s. Relative expression was calculated by the 2^−∆∆CT^ method. For all quantitative analyses, we performed three biological replicates and three technical replicates. An unpaired t-test was used to analyze the significance of each tissue of the Ma bamboo. Genes were considered signifificantly deregulated when the fold change was ≥1.5 and *p*-value < 0.05.

## 3. Results

### 3.1. MIKCc-Type MADS-Box Genes Identification and Phylogenetic Tree Construction

**Figure 1:**
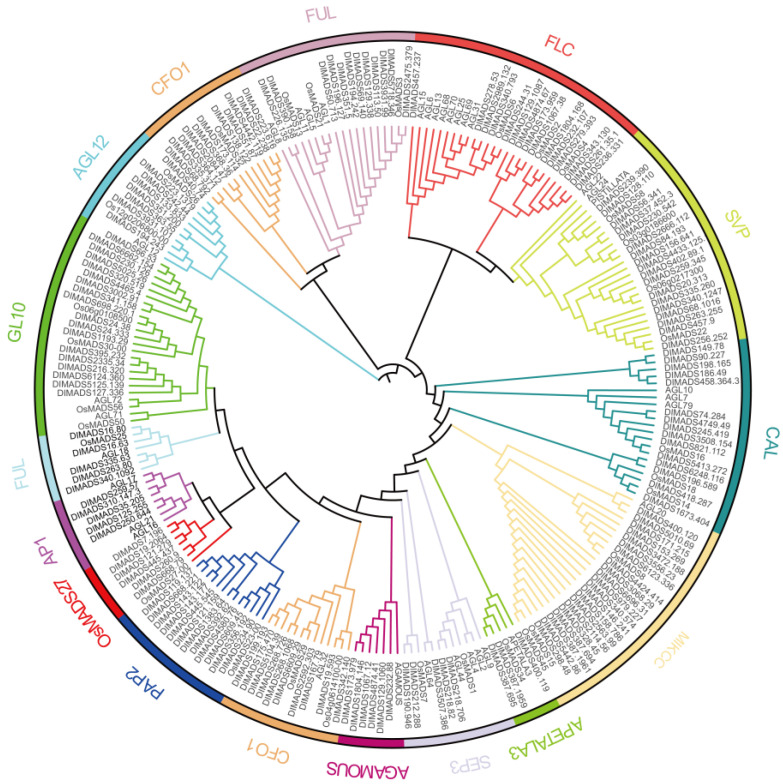
Phylogenetic analysis of MIKCc-type MADS-box proteins in *D. latiflorus*. A neighbor-joining (NJ) tree was constructed using 170 MADS-box sequence. The phylogenetic tree is clustered in 15 subfamilies, which are shown in different colors.

A total of 170 coding sequences were identified from *D. latiflorus* genome-wide were used in the HMMER search program and named DiMADS01 to DiMADS170 for the convenience of classification (Table S2). In order to classify these genes, we used a protein sequence in the MEGA program for the alignment of the phylogenetic tree (Figure 1). The MIKCc-type MADS-box gene is divided into 15 subfamilies. The two largest clades contain 18 members belonging to the SVP and GL10 subfamilies. In contrast, the APETALA3 subfamily contains only three members. In addition, 21 MIKCc-type MADS-box genes have not yet been classified into any subfamily.

### 3.2. Hormone-Related Promoter Cis-Regulatory Elements

Phytohormone regulation plays an influential role in plant flowering. We searched 1500 bp upstream of the MIKCc-type MADS-box gene. A total of 1137 *cis*-acting elements related to phytohormone associations were identified, covering 162 genes (Figure S1). In these *cis*-acting genes, auxin, abscisic acid, gibberellin and jasmonic acid were found at 79, 476, 96, 486 sites and cover 61, 103, 73, 128 genes. In addition, individual genetic differences are significant. For example, the genes DIMADS1383.64 and DIMADS236.331 have only one phytohormone-related *cis*-acting element, while DIMADS387.1961 has 12.

### 3.3. Synteny Analysis between D. latiflflorus and O. sativa

**Figure 2:**
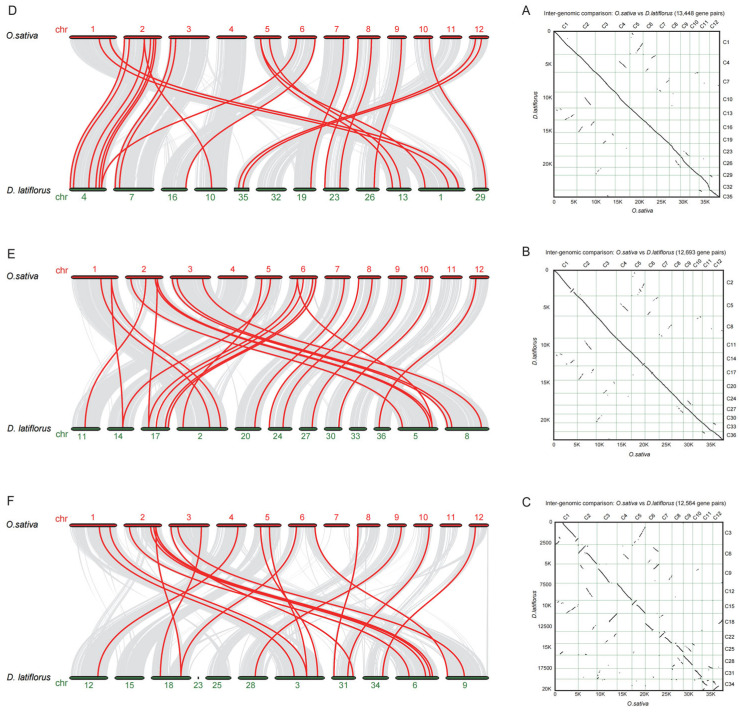
The synteny relationship between *D. latiflorus* and *O. sativa*. (**A**–**C**): MIKCc-type MADS-box gene synteny between *D. latiflorus* subgenome A, B, C and rice. (**D**–**F**): genome synteny between subgenome A, B, C of *D. latiflorus* and *O. sativa*.

First, we used MCscanX software to analyze and obtain high genome synteny between rice and bamboo. Visual result showed that subgenomes A and B are better than subgenomes C (Figure 2A–C). According to the isomorphism results of the MIKCc-type MADS-box gene synteny (Table S3), subgenome A, subgenome A’, subgenome B, subgenome B’, subgenome C and subgenome C’ contain 24, 25, 22, 18, 18 and 21 family genes (Figure 2D–F; Figure S2), respectively. In the analysis of the gene locations of bamboo chromosomes, it was found that chromosome 4 had the largest distribution, with a total of 7 genes, while chromosomes 32, 33, 15, 23 and 25 had no isogene distribution. A total of 39 bamboo genes were missing from the analysis. The results demonstrate that the number of MIKCc-type MADS-box gene in each 12 chomosomes of bamboo was significantly lower than that of rice (75MIKCc-type MADS-box gene).

### 3.4. Transcriptome-Scale Analysis of Floral Organ

**Figure 3:**
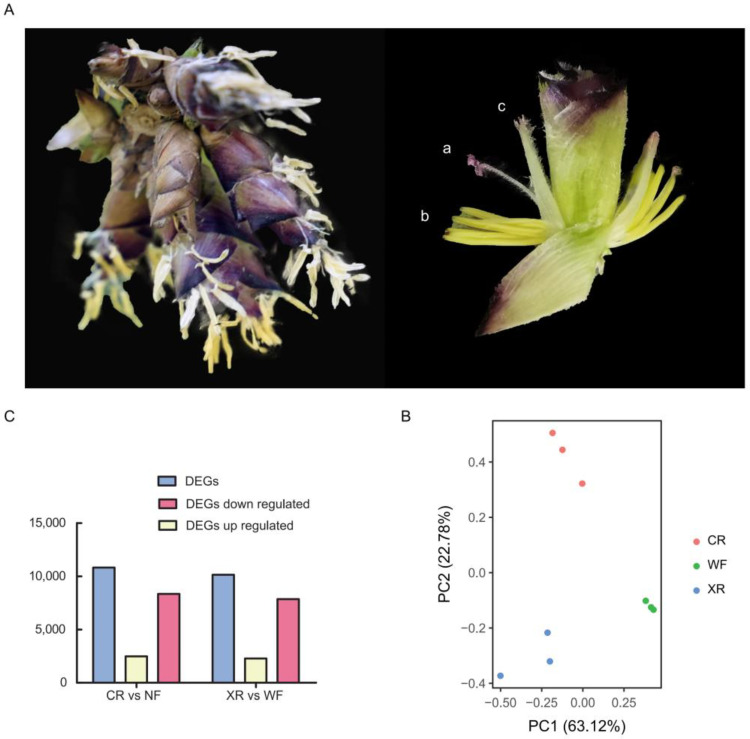
Materials used for transcriptome sequencing and data quality assessment. (**A**) *D. latiflorus* flower organ; (a) pistil; (b) stamen; (c) palea. (**B**) Multi-dimensional scaling (MDS) plots to analyze the variation among flower organ tissues. (**C**) Multi-dimensional scaling (MDS) plots to analyze the variation among flower organ tissues.

A total of 9 cDNA libraries were constructed using total RNA from pistil, stamen, and palea (Figure 3A). A total of 22973946, 21152812 and 21786587 reads were obtained to follow these tissues, respectively. Each database has a effective rate above 98% (Table S4). We generated multi-dimensional scaling (MDS) diagrams to illustrate the similarities between repeats and organizations (Figure 3B). We found that all the stages were clearly dispersed and biologically repeatedly approached each other. Among the differentially expressed genes (DEGs), there were 10,821 differentially expressed genes detected in pistil compared to CK, including 2476 that were upregulated and 8345 that were downregulated genes; Compared with the stamen, CK detected 10,821 differentially expressed genes, 2285 upregulated and 7862 downregulated genes (Figure 3C).

### 3.5. Go Enrichment Analysis

**Figure 4:**
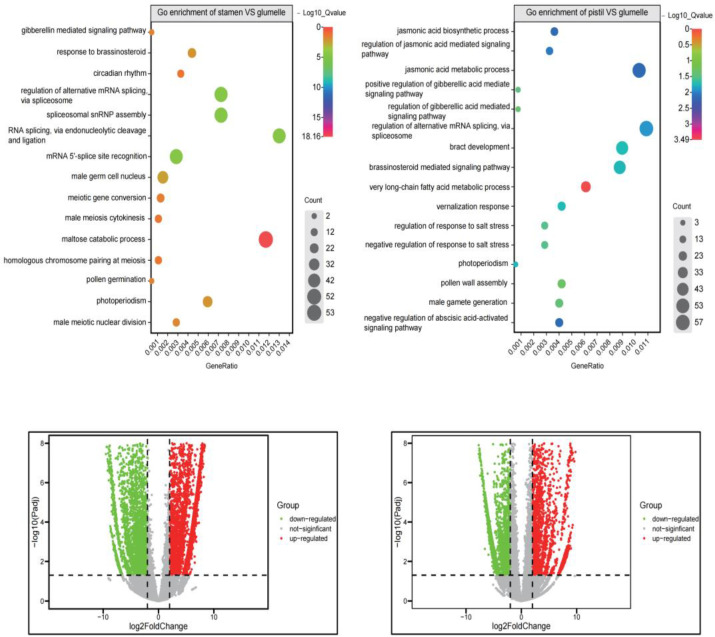
Gene ontology enrichment analysis of DEGs in flower organs.

The molecular mechanisms underlying the flowering of Ma bamboo and the small amount of pollen remains a mystery. A pair-wise comparison of pistil vs. CK and stamen vs. CK showed that the gene ontology terms are overrepresented among the DEGs (Figure 4), including those related to the flowering process, such as photoperiod, circadian rhythm and vernalization response. Thus, these DEGs may be the basis of Ma bamboo blooms. Homology-related terms include the positive regulation of gibberellin-mediated signaling pathways, brassosteroid-mediated signaling pathways and the jasmonic acid biosynthetic process. These DEGs were also enriched in Ma bamboo, suggesting that hormones may regulate the Ma bamboo flowering. The functional terms related to biological stress enriched the regulation of salt stress response and the negative regulation of abscisic-acid-activated signaling pathway. This could mean that Ma bamboo has the potential to bloom under stress. Interestingly, jasmonic acid biosynthetic process and jasmonic acid metabolic process were enriched in pistils. Studies have shown that jasmonic acid has an inhibitory effect on pollen formation, which may be the reason for the low pollen count of Ma bamboo.

### 3.6. Expression of MIKCc-Type MADS-Box Gene in Flower Organs

**Figure 5:**
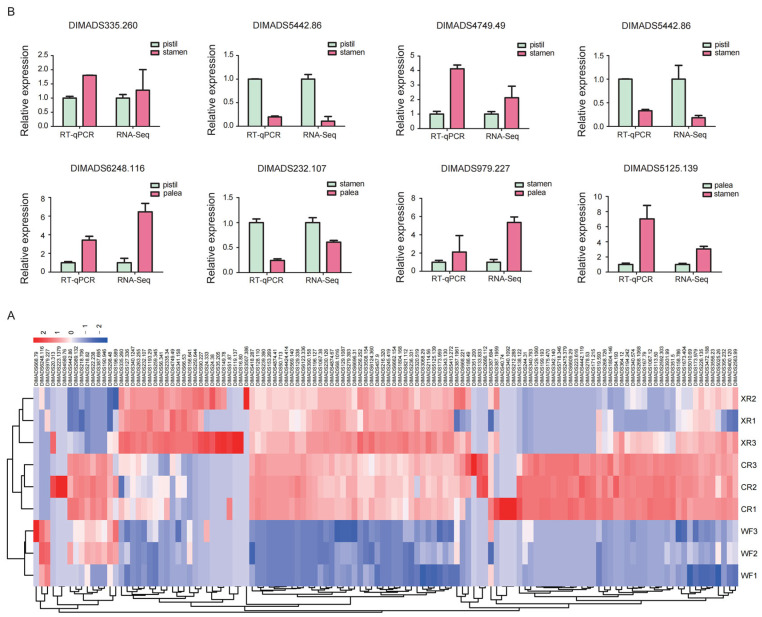
Expression of MIKCc-type MADS-box gene in flower organs. (**A**) MADS-box genes obtained by RT-qPCR analysis. (**B**) Heatmap of the expression of 170 MIKCc-type MADS-box genes in the flower organ.

During flower development, we performed RNA-seq analysis of 170 MIKCc-type MADS-box gene expression patterns (Figure 5A). These genes were highly expressed in the flower organs compared to the control. In the pistil and stamen, the GL10 subfamily, AP1 (A model) subfamily, and SVP subfamily were significantly highly expressed in the stamens, while CFO subfamily, AGAMOUS (C model) subfamily, and FUL subfamily were significantly highly expressed in the pistil. Interestingly, GL10 regulates seed weight by affecting the gibberellin signaling pathway, which correlates with the results of our enrichment of DEGs in the gibberellin metabolic pathway. This result may indicate that the stamens are more sensitive to gibberellin signaling. CFO is an evolutionary branch unique to Poaceae plants that determines the development of stamens, pistils, and ovules. In addition, the expression of DIMADS668.79, DIMADS6248.116, DIMADS979.227, DIMADS20.313, and DIMADS223.1379 were lower in the pistil and stamen but higher in CK. To verify the RNA-seq results, we selected 11 genes for further confirmation by RT-qPCR. The expression profiles of eight genes (Figure 5B) were consistent with the results of RNA-seq, indicating that the data of RNA-seq were reliable.

## 4. Discussion

Most woody bamboos are polyploid species. They have certain advantages in the number of flowering genes but also have flower-negative traits [5]. The published genome of the Ma bamboo paved a way for solving the mystery of Ma bamboo flowering. With the establishment of a gene-editing system, Ma bamboo is expected to become a model plant of Bambusoideae; therefore, it is necessary to study the flowering genes and flower organ gene expression.

In this study, 170 MIKCc-type MADS-box genes were identified and divided into 15 branches. The number of MIKCc-type MADS-box genes in most flowering plants is between 40 and 70 [33]. For example, *O. sativa* and *Arabidopsis thaliana* have similar populations (43 and 45, respectively) [34,35]. However, we found that the content of bamboo was significantly higher than that of rice. This is partly the result of hexaploidy, and similar results were found in wheat with 201 genes [36]. In addition, rice has 43 MIKCc-type MADS-box genes per 12 chromosomes, while Ma bamboo has less than 27, which is much less proportionally. Whether this means that there are insufficient functional genes for flowering is unknown.

Stable auxin signals enables a homogeneous flower size, while variations in JA and ABA signals may be responsible for flower numbers [37]. Floral organs are the result of the coordinated expression of several genes to form sepals, petals, stamens, and carpels, whose identity is established by a combination of MADS-box genes [38,39]. Previous studies have shown that hormones are important factors mediating the bamboo flowering process [19,40]. We found 1137 phytohormone-associated *cis*-acting elements and covering 162 MIKCc-type MADS-box genes. This suggests that most genes can be regulated by hormones. Various phytohormone-related pathways, such as gibberellin-related metabolic pathways, have been enriched from DEGs under comparative transcriptome analysis. A previous study revealed that GA induces MADS-box gene expression [20], and high gibberellin was found in bamboo flower under drought stress [41,42]. These results prove that hormones are crucial in bamboo blooms. Furthermore, we enriched GO terms about vernalization and circadian rhythm-related pathways. These stable biological clocks may not be the key reason for bamboo’s irregular flowering.

We found that a large number of jasmonic acid *cis*-acting elements and a large number of DEGs were enriched in the jasmonic acid metabolic pathway in stamens. Interestingly, jasmonic acid has been shown to inhibit pollen formation [43,44]. This may explain why bamboo has more flowers but low pollen and seed setting rates.

We found that the GL10 and SVP subfamily genes were highly expressed in the pistil. In rice, GL10 encodes the MADS-box family transcription factor OsMADS56, which positively regulates rice seed weight by affecting the gibberellin signaling pathway [45]. In contrast, SVP (Short Vegetative Phase) directly acts on the cytochrome P450 mono-oxygenase CYP707A1/CYP707A3 and glucosidase AtBG1 in *Arabidopsis* to control the content of blatoacid, thus resisting drought stress in plants [46]. This suggests that the stamens are more likely regulated by hormones. Combined with the concentration associated with drought stress in the GO term of the stamens, it was possible that drought was affecting the flowering of these bamboo plants.

## 5. Conclusions

This is the first identification and characterization of MIKCc-type MADS-box, the main flowering-related gene family in *D. latiflorus*. A total of 170 family members were recognized, which can be divided into 12 subfamilies. Genome synteny analysis of Ma bamboo compared with rice shows that subgenomes AA and BB are better than CC and obtained 49, 40, 39 synteny genes. There are a large number of MIKCc-type MADS-box family genes in *D. latiflorus*, and the high expression of these genes in stamen and pistil proves that they participate in the flowering process. Moreover, in transcriptome analysis, a large number of differential genes related to hormones, such as gibberellin and jasmonic acid, were enriched, indicating that hormones regulate flowering. The concentration of jasmonic acid in the pistil may be the reason why the pistil has less pollen. Taken together, MIKCc-type MADS-box genes may be engaged in the flowering processes in *D. latiflorus*. Our results will provide more evidence for the flowering mechanism of Ma bamboo for future studies.

## Data Availability

Not applicable.

## Supplementary Materials

The following supporting information can be downloaded at: https://www.mdpi.com/article/10.3390/genes14010078/s1, Table S1. Primer sequences for RT-PCR; Table S2. List of all sequences identified by PFAM domain (type II); Table S3. List of synteny genes of MIKCc-type MADS-box genes between rice and bamboo; Table S4. Summary of the quality of transcriptome sequencing data for floral tissue; Figure S1. The synteny relationship between *D. latiflorus* and rice. (A)(B)(C)MIKCc-type MADS-box gene synteny between *D. latiflorus* subgenome A’, B’, C’ and rice; Figure S2. Hormone-associated *cis*-acting elements in MIKCc-type MADS-box genes.
