# Supplementary material for: Genome-Wide Identification of MIKCc-Type MADS-Box Family Gene and Floral Organ Transcriptome Characterization in Ma Bamboo (Dendrocalamus latiflorus Munro)
Source: Genes (Basel). 2022 Dec 27;14(1):78. doi: 10.3390/genes14010078 (PMC9859424; doi:10.3390/genes14010078)
Supplement: Supplementary file 1 [file genes-14-00078-s001.zip › genes-2086018-supplementary.pdf]

Table S1. Primer sequences for RT-PCR.

Primer Name	Primer Sequence	Gene Name
DIMADS335.260_1F	5′-CGCTCATCGTCTTCTCCTCC-3′	evm.model.FRAGSCAFF_335.260
DIMADS335.260_R	5′-TGCATGTTCTCCTCTGCCAG-3′	evm.model.FRAGSCAFF_335.260
DIMADS5442.86_1F	5′-AGGGAAATTGGTGCCACGAA-3′	evm.model.ORIGINAL_5442.86
DIMADS5442.86_R	5′- TTGAGTTTGGGCCTGGGTTT-3′	evm.model.ORIGINAL_5442.86
DIMADS4749.49_1F	5′-AGTACCACGAGTTCTGCAGC-3′	evm.model.ORIGINAL_4749.49
DIMADS4749.49_R	5′- TGTATGCCTCGTACGAGTGC-3′	evm.model.ORIGINAL_4749.49
DIMADS5442.86_1F	5′-AGGGAAATTGGTGCCACGAA-3′	evm.model.ORIGINAL_5442.86
DIMADS5442.86_R	5′- TTGAGTTTGGGCCTGGGTTT-3′	evm.model.ORIGINAL_5442.86
DIMADS6248.116_1F	5′-GCCATGATGTTGGGCCTAGT-3′	evm.model.ORIGINAL_6248.116
DIMADS6248.116_R	5′- TCACTGGCCACAACAACTGT-3′	evm.model.ORIGINAL_6248.116
DIMADS232.107_1F	5′-GGATCGAGGACCGGACGA-3′	evm.model.FRAGSCAFF_232.107
DIMADS232.107_R	5′- CCCCTAGGACGACTGGAGTT-3′	evm.model.FRAGSCAFF_232.107
DIMADS979.227_1F	5′-AAGAAGGCGCACGAGATCTC-3′	evm.model.ORIGINAL_979.227
DIMADS979.227_R	5′- TCCCATGAGGTGCTTGTGAC-3′	evm.model.ORIGINAL_979.227
DIMADS5125.139_1F	5′-GAAGAGCCTCCACAACGTCA-3′	evm.model.ORIGINAL_5125.139
DIMADS5125.139_R	5′- TGTAGAGCTCCGTCTCCACA-3′	evm.model.ORIGINAL_5125.139

Table S2. List of all sequences identified by PFAM domain (type II).

Gene-ID	Clade	Pfam-IDs	New Gene name
evm.model.FRAGSCAFF_256.252	SVP	PF00319	DIMADS256.252
evm.model.FRAGSCAFF_457.9	SVP	PF00319	DIMADS457.9
evm.model.FRAGSCAFF_263.255	SVP	PF00319	DIMADS263.255
evm.model.FRAGSCAFF_335.63	SVP	PF00319	DIMADS335.63
evm.model.FRAGSCAFF_68.1016	SVP	PF00319	DIMADS68.1016
evm.model.FRAGSCAFF_340.1247	SVP	PF00319	DIMADS340.1247
evm.model.FRAGSCAFF_335.260	SVP	PF00319	DIMADS335.260
evm.model.FRAGSCAFF_20.313	SVP	PF00319	DIMADS20.313
evm.model.FRAGSCAFF_259.345	SVP	PF00319	DIMADS259.345
evm.model.FRAGSCAFF_402.89.1	SVP	PF00319	DIMADS402.89.1
evm.model.FRAGSCAFF_156.641	SVP	PF00319	DIMADS156.641
evm.model.FRAGSCAFF_84.193	SVP	PF00319	DIMADS84.193
evm.model.FRAGSCAFF_2666.112	SVP	PF00319	DIMADS2666.112
evm.model.FRAGSCAFF_230.542	SVP	PF00319	DIMADS230.542
evm.model.FRAGSCAFF_37.452.3	SVP	PF00319	DIMADS37.452.3
evm.model.FRAGSCAFF_56.341	SVP	PF00319	DIMADS56.341
evm.model.FRAGSCAFF_128.110	SVP	PF00319	DIMADS128.110
evm.model.ORIGINAL_4433.125.1	SVP	PF00319	DIMADS4433.125.1
evm.model.FRAGSCAFF_190.946	SPE3	PF00319	DIMADS190.946
evm.model.FRAGSCAFF_212.288	SPE3	PF00319	DIMADS212.288
evm.model.FRAGSCAFF_3507.386	SPE3	PF00319	DIMADS3507.386
evm.model.FRAGSCAFF_218.82	SPE3	PF00319	DIMADS218.82
evm.model.FRAGSCAFF_218.706	SPE3	PF00319	DIMADS218.706
evm.model.FRAGSCAFF_119.137	PAP2	PF00319	DIMADS119.137
evm.model.FRAGSCAFF_143.122	PAP2	PF00319	DIMADS143.122
evm.model.FRAGSCAFF_143.177	PAP2	PF00319	DIMADS143.177
evm.model.FRAGSCAFF_445.545	PAP2	PF00319	DIMADS445.545
evm.model.FRAGSCAFF_121.159	PAP2	PF00319	DIMADS121.159
evm.model.FRAGSCAFF_392.62	PAP2	PF00319	DIMADS392.62
evm.model.FRAGSCAFF_356.192	PAP2	PF00319	DIMADS356.192
evm.model.FRAGSCAFF_234.90	PAP2	PF00319	DIMADS234.90
evm.model.ORIGINAL_668.221	PAP2	PF00319	DIMADS668.221
evm.model.ORIGINAL_1383.64	PAP2	PF00319	DIMADS1383.64
evm.model.ORIGINAL_4580.76	PAP2	PF00319	DIMADS4580.76
evm.model.ORIGINAL_609.45	PAP2	PF00319	DIMADS609.45
evm.model.FRAGSCAFF_19.2064	OSMADS27	PF00319	DIMADS19.2064
evm.model.FRAGSCAFF_121.279	OSMADS27	PF00319	DIMADS121.279
evm.model.FRAGSCAFF_445.437	OSMADS27	PF00319	DIMADS445.437
evm.model.FRAGSCAFF_260.9	OSMADS27	PF00319	DIMADS260.9
evm.model.FRAGSCAFF_7.196	OSMADS27	PF00319	DIMADS7.196
evm.model.ORIGINAL_668.79	OSMADS27	PF00319	DIMADS668.79
evm.model.FRAGSCAFF_298.48	MIKCC	PF00319	DIMADS298.48

evm.model.FRAGSCAFF_387.1961	MIKCC	PF00319	DIMADS387.1961
evm.model.FRAGSCAFF_387.694	MIKCC	PF00319	DIMADS387.694
evm.model.FRAGSCAFF_328.45	MIKCC	PF00319	DIMADS328.45
evm.model.FRAGSCAFF_158.786	MIKCC	PF00319	DIMADS158.786
evm.model.FRAGSCAFF_146.241	MIKCC	PF00319	DIMADS146.241
evm.model.FRAGSCAFF_340.574	MIKCC	PF00319	DIMADS340.574
evm.model.FRAGSCAFF_242.44	MIKCC	PF00319	DIMADS242.44
evm.model.FRAGSCAFF_4424.414 0	MIKCC	PF00319	DIMADS4424.414 0
evm.model.FRAGSCAFF_6123.336	MIKCC	PF00319	DIMADS6123.336
evm.model.FRAGSCAFF_3472.188 3	MIKCC	PF00319	DIMADS3472.188 3
evm.model.FRAGSCAFF_153.269	MIKCC	PF00319	DIMADS153.269
evm.model.FRAGSCAFF_171.215	MIKCC	PF00319	DIMADS171.215
evm.model.FRAGSCAFF_400.120	MIKCC	PF00319	DIMADS400.120
evm.model.ORIGINAL_5442.86	MIKCC	PF00319	DIMADS5442.86
evm.model.ORIGINAL_2114.56	MIKCC	PF00319	DIMADS2114.56
evm.model.ORIGINAL_2563.99	MIKCC	PF00319	DIMADS2563.99
evm.model.ORIGINAL_979.227	MIKCC	PF00319	DIMADS979.227
evm.model.ORIGINAL_6696.31	MIKCC	PF00319	DIMADS6696.31
evm.model.ORIGINAL_3068.29	MIKCC	PF00319	DIMADS3068.29
evm.model.ORIGINAL_3556.23	MIKCC	PF00319	DIMADS3556.23
evm.model.ORIGINAL_5010.69	MIKCC	PF00319	DIMADS5010.69
evm.model.FRAGSCAFF_66.53	GL10	PF00319	DIMADS66.53
evm.model.FRAGSCAFF_6662.154	GL10	PF00319	DIMADS6662.154
evm.model.FRAGSCAFF_230.126	GL10	PF00319	DIMADS230.126
evm.model.FRAGSCAFF_320.519	GL10	PF00319	DIMADS320.519
evm.model.FRAGSCAFF_341.158	GL10	PF00319	DIMADS341.158
evm.model.FRAGSCAFF_698.220.1	GL10	PF00319	DIMADS698.220.1
evm.model.FRAGSCAFF_24.38	GL10	PF00319	DIMADS24.38
evm.model.FRAGSCAFF_24.333	GL10	PF00319	DIMADS24.333
evm.model.FRAGSCAFF_395.232	GL10	PF00319	DIMADS395.232
evm.model.FRAGSCAFF_216.320	GL10	PF00319	DIMADS216.320
evm.model.FRAGSCAFF_6124.360	GL10	PF00319	DIMADS6124.360
evm.model.ORIGINAL_5025.26	GL10	PF00319	DIMADS5025.26
evm.model.ORIGINAL_4465.4	GL10	PF00319	DIMADS4465.4
evm.model.ORIGINAL_3002.91	GL10	PF00319	DIMADS3002.91
evm.model.ORIGINAL_1193.29	GL10	PF00319	DIMADS1193.29
evm.model.ORIGINAL_2335.34	GL10	PF00319	DIMADS2335.34
evm.model.ORIGINAL_5125.139	GL10	PF00319	DIMADS5125.139
evm.model.FRAGSCAFF_16.80	FUL	PF00319	DIMADS16.80
evm.model.FRAGSCAFF_16.63	FUL	PF00319	DIMADS16.63
evm.model.FRAGSCAFF_335.63	FUL	PF00319	DIMADS335.63
evm.model.FRAGSCAFF_263.80	FUL	PF00319	DIMADS263.80
evm.model.FRAGSCAFF_340.1092	FUL	PF00319	DIMADS340.1092
evm.model.FRAGSCAFF_2475.379	FUL	PF00319	DIMADS2475.379

evm.model.FRAGSCAFF_5718.346	FUL	PF00319	DIMADS5718.346
evm.model.FRAGSCAFF_113.50	FUL	PF00319	DIMADS113.50
evm.model.FRAGSCAFF_129.338	FUL	PF00319	DIMADS129.338
evm.model.FRAGSCAFF_194.242	FUL	PF00319	DIMADS194.242
evm.model.FRAGSCAFF_351.5	FUL	PF00319	DIMADS351.5
evm.model.FRAGSCAFF_196.127	FUL	PF00319	DIMADS196.127
evm.model.FRAGSCAFF_50.713	FUL	PF00319	DIMADS50.713
evm.model.FRAGSCAFF_390.158	FUL	PF00319	DIMADS390.158
evm.model.FRAGSCAFF_226.135	FUL	PF00319	DIMADS226.135
evm.model.ORIGINAL_3931.99	FUL	PF00319	DIMADS3931.99
evm.model.ORIGINAL_569.140	FUL	PF00319	DIMADS569.140
evm.model.FRAGSCAFF_236.331	FLC	PF00319	DIMADS236.331
evm.model.FRAGSCAFF_261.35.1	FLC	PF00319	DIMADS261.35.1
evm.model.FRAGSCAFF_343.130	FLC	PF00319	DIMADS343.130
evm.model.FRAGSCAFF_279.393	FLC	PF00319	DIMADS279.393
evm.model.FRAGSCAFF_232.107	FLC	PF00319	DIMADS232.107
evm.model.FRAGSCAFF_1804.168	FLC	PF00319	DIMADS1804.168
evm.model.FRAGSCAFF_173.959	FLC	PF00319	DIMADS173.959
evm.model.FRAGSCAFF_129.1087	FLC	PF00319	DIMADS129.1087
evm.model.FRAGSCAFF_244.31	FLC	PF00319	DIMADS244.31
evm.model.FRAGSCAFF_340.793	FLC	PF00319	DIMADS340.793
evm.model.FRAGSCAFF_278.53	FLC	PF00319	DIMADS278.53
evm.model.FRAGSCAFF_457.237	FLC	PF00319	DIMADS457.237
evm.model.ORIGINAL_1383.64	FLC	PF00319	DIMADS1383.64
evm.model.ORIGINAL_1067.38	FLC	PF00319	DIMADS1067.38
evm.model.ORIGINAL_4874.67	FLC	PF00319	DIMADS4874.67
evm.model.FRAGSCAFF_199.193	CFO1	PF00319	DIMADS199.193
evm.model.FRAGSCAFF_175.470	CFO1	PF00319	DIMADS175.470
evm.model.FRAGSCAFF_268.726	CFO1	PF00319	DIMADS268.726
evm.model.FRAGSCAFF_268.1068	CFO1	PF00319	DIMADS268.1068
evm.model.FRAGSCAFF_2592.303	CFO1	PF00319	DIMADS2592.303
evm.model.FRAGSCAFF_167.79	CFO1	PF00319	DIMADS167.79
evm.model.FRAGSCAFF_19.593	CFO1	PF00319	DIMADS19.593
evm.model.FRAGSCAFF_342.140	CFO1	PF00319	DIMADS342.140
evm.model.FRAGSCAFF_223.616	CFO1	PF00319	DIMADS223.616
evm.model.FRAGSCAFF_22.238	CFO1	PF00319	DIMADS22.238
evm.model.FRAGSCAFF_51.87	CFO1	PF00319	DIMADS51.87
evm.model.FRAGSCAFF_138.122	CFO1	PF00319	DIMADS138.122
evm.model.FRAGSCAFF_173.114	CFO1	PF00319	DIMADS173.114
evm.model.FRAGSCAFF_368.36	CFO1	PF00319	DIMADS368.36
evm.model.FRAGSCAFF_364.12	CFO1	PF00319	DIMADS364.12
evm.model.FRAGSCAFF_335.371	CFO1	PF00319	DIMADS335.371
evm.model.FRAGSCAFF_68.792	CFO1	PF00319	DIMADS68.792
evm.model.ORIGINAL_5104.39	CFO1	PF00319	DIMADS5104.39

evm.model.ORIGINAL_6609.29	CFO1	PF00319	DIMADS6609.29
evm.model.ORIGINAL_4442.119	CFO1	PF00319	DIMADS4442.119
evm.model.ORIGINAL_2084.47	CFO1	PF00319	DIMADS2084.47
evm.model.FRAGSCAFF_1673.404	CAL	PF00319	DIMADS1673.404
evm.model.FRAGSCAFF_418.287	CAL	PF00319	DIMADS418.287
evm.model.FRAGSCAFF_196.589	CAL	PF00319	DIMADS196.589
evm.model.FRAGSCAFF_5413.272	CAL	PF00319	DIMADS5413.272
evm.model.FRAGSCAFF_821.112	CAL	PF00319	DIMADS821.112
evm.model.FRAGSCAFF_245.419	CAL	PF00319	DIMADS245.419
evm.model.FRAGSCAFF_74.284	CAL	PF00319	DIMADS74.284
evm.model.FRAGSCAFF_458.364.3	CAL	PF00319	DIMADS458.364.3
evm.model.FRAGSCAFF_186.49	CAL	PF00319	DIMADS186.49
evm.model.FRAGSCAFF_198.165	CAL	PF00319	DIMADS198.165
evm.model.FRAGSCAFF_90.227	CAL	PF00319	DIMADS90.227
evm.model.FRAGSCAFF_149.78	CAL	PF00319	DIMADS149.78
evm.model.ORIGINAL_6248.116	CAL	PF00319	DIMADS6248.116
evm.model.ORIGINAL_3508.154	CAL	PF00319	DIMADS3508.154
evm.model.ORIGINAL_4749.49	CAL	PF00319	DIMADS4749.49
evm.model.FRAGSCAFF_387.695	APETALA3	PF00319	DIMADS387.695
evm.model.FRAGSCAFF_387.1959	APETALA3	PF00319	DIMADS387.1959
evm.model.FRAGSCAFF_400.119	APETALA3	PF00319	DIMADS400.119
evm.model.FRAGSCAFF_259.57	AP1	PF00319	DIMADS259.57
evm.model.FRAGSCAFF_310.147.3	AP1	PF00319	DIMADS310.147.3
evm.model.FRAGSCAFF_35.205	AP1	PF00319	DIMADS35.205
evm.model.FRAGSCAFF_125.255	AP1	PF00319	DIMADS125.255
evm.model.FRAGSCAFF_250.644	AP1	PF00319	DIMADS250.644
evm.model.FRAGSCAFF_40.74	AGL12	PF00319	DIMADS40.74
evm.model.FRAGSCAFF_223.1379	AGL12	PF00319	DIMADS223.1379
evm.model.FRAGSCAFF_242.44	AGL12	PF00319	DIMADS242.44
evm.model.FRAGSCAFF_133.833	AGL12	PF00319	DIMADS133.833
evm.model.FRAGSCAFF_351.200	AGL12	PF00319	DIMADS351.200
evm.model.FRAGSCAFF_363.55	AGL12	PF00319	DIMADS363.55
evm.model.FRAGSCAFF_3931.101	AGL12	PF00319	DIMADS3931.101
evm.model.FRAGSCAFF_194.243	AGL12	PF00319	DIMADS194.243
evm.model.FRAGSCAFF_173.979	AGAMOUS	PF00319	DIMADS173.979
evm.model.FRAGSCAFF_4874.41	AGAMOUS	PF00319	DIMADS4874.41
evm.model.FRAGSCAFF_129.1060	AGAMOUS	PF00319	DIMADS129.1060
evm.model.FRAGSCAFF_232.88	AGAMOUS	PF00319	DIMADS232.88
evm.model.ORIGINAL_1804.146	AGAMOUS	PF00319	DIMADS1804.146
evm.model.ORIGINAL_1067.12	AGAMOUS	PF00319	DIMADS1067.12

Table S3. List of synteny genes of MIKCc-type MADS-box genes between rice and bamboo.

Oryza sativa Gene-ID	D. latiflorusGene-ID	subgenome	New Gene name
Os02t0682200-01	evm.TU.FRAGSCAFF_278.53	subgenomeA1	DlMADS278.53
Os02t0579600-00	evm.TU.FRAGSCAFF_250.644	subgenomeA1	DlMADS250.644
Os02t0579600-00	evm.TU.FRAGSCAFF_19.2064	subgenomeA1	DlMADS19.2064
Os01t0886200-01	evm.TU.FRAGSCAFF_129.1060	subgenomeA1	DlMADS129.1060
Os07t0605200-01	evm.TU.ORIGINAL_2563.99	subgenomeA1	DlMADS2563.99
Os05t0203800-01	evm.TU.FRAGSCAFF_128.110	subgenomeA1	DlMADS128.110
Os05t0203800-01	evm.TU.FRAGSCAFF_226.135	subgenomeA1	DlMADS226.135
Os09t0507200-01	evm.TU.FRAGSCAFF_171.215	subgenomeA1	DlMADS171.215
Os12t0207000-01	evm.TU.FRAGSCAFF_113.50	subgenomeA1	DlMADS113.50
Os02t0104100-00	evm.TU.FRAGSCAFF_24.38	subgenomeA1	DlMADS24.38
Os02t0731200-01	evm.TU.FRAGSCAFF_335.371	subgenomeA1	DlMADS335.371
Os03t0122600-01	evm.TU.FRAGSCAFF_230.126	subgenomeA1	DlMADS230.126
Os02t0170300-01	evm.TU.FRAGSCAFF_268.726	subgenomeA1	DlMADS268.726
Os08t0431900-00	evm.TU.FRAGSCAFF_356.192	subgenomeA1	DlMADS356.192
Os06t0712700-01	evm.TU.ORIGINAL_821.112	subgenomeA1	DlMADS821.112
Os08t0112700-01	evm.TU.FRAGSCAFF_363.55	subgenomeA1	DlMADS363.55
Os10t0536100-01	evm.TU.FRAGSCAFF_395.232	subgenomeA1	DlMADS395.232
Os12t0206800-00	evm.TU.FRAGSCAFF_113.52	subgenomeA1	DlMADS113.52
Os12t0501700-00	evm.TU.FRAGSCAFF_149.78	subgenomeA1	DlMADS149.78
Os02t0761000-01	evm.TU.FRAGSCAFF_256.252	subgenomeA1	DlMADS256.252
Os01t0726400-01	evm.TU.FRAGSCAFF_223.616	subgenomeA1	DlMADS223.616
Os01t0883100-01	evm.TU.FRAGSCAFF_129.1087	subgenomeA1	DlMADS129.1087
Os05t0423400-01	evm.TU.FRAGSCAFF_236.331	subgenomeA1	DlMADS236.331
Os03t0186600-01	evm.TU.FRAGSCAFF_230.542	subgenomeA1	DlMADS230.542
Os02t0682200-01	evm.TU.ORIGINAL_2989.132	subgenomeA2	DlMADS2989.132
Os02t0579600-00	evm.TU.ORIGINAL_3507.385	subgenomeA2	DlMADS3507.385
Os02t0579600-00	evm.TU.FRAGSCAFF_7.196	subgenomeA2	DlMADS7.196
Os01t0886200-01	evm.TU.ORIGINAL_1067.12	subgenomeA2	DlMADS1067.12
Os07t0605200-01	evm.TU.ORIGINAL_6696.31	subgenomeA2	DlMADS6696.31
Os05t0203800-01	evm.TU.FRAGSCAFF_239.390	subgenomeA2	DlMADS239.390
Os05t0203800-01	evm.TU.FRAGSCAFF_390.158	subgenomeA2	DlMADS390.158
Os09t0507200-01	evm.TU.ORIGINAL_4424.414	subgenomeA2	DlMADS4424.414
Os12t0207000-01	evm.TU.ORIGINAL_3931.99	subgenomeA2	DlMADS3931.99
Os02t0104100-00	evm.TU.FRAGSCAFF_24.333	subgenomeA2	DlMADS24.333
Os02t0731200-01	evm.TU.FRAGSCAFF_335.63	subgenomeA2	DlMADS335.63
Os02t0731200-01	evm.TU.FRAGSCAFF_35.205	subgenomeA2	DlMADS35.205
Os03t0122600-01	evm.TU.ORIGINAL_6662.154	subgenomeA2	DlMADS6662.154
Os02t0170300-01	evm.TU.FRAGSCAFF_268.1068	subgenomeA2	DlMADS268.1068
Os08t0431900-00	evm.TU.FRAGSCAFF_234.90	subgenomeA2	DlMADS234.90
Os06t0712700-01	evm.TU.ORIGINAL_3508.154	subgenomeA2	DlMADS3508.154
Os08t0112700-01	evm.TU.FRAGSCAFF_351.200	subgenomeA2	DlMADS351.200

Os10t0536100-01	evm.TU.FRAGSCAFF_216.320	subgenomeA2	DIMADS216.320
Os12t0206800-00	evm.TU.ORIGINAL_3931.101	subgenomeA2	DIMADS3931.101
Os12t0501700-00	evm.TU.FRAGSCAFF_90.227	subgenomeA2	DIMADS90.227
Os06t0217300-01	evm.TU.FRAGSCAFF_335.260	subgenomeA2	DIMADS335.260
Os04t0614100-00	evm.TU.FRAGSCAFF_19.593	subgenomeA2	DIMADS19.593
Os01t0726400-01	evm.TU.FRAGSCAFF_22.238	subgenomeA2	DIMADS22.238
Os05t0423400-01	evm.TU.ORIGINAL_1067.38	subgenomeA2	DIMADS1067.38
Os03t0186600-01	evm.TU.ORIGINAL_2666.112	subgenomeA2	DIMADS2666.112
Os02t0579600-00	evm.TU.ORIGINAL_668.79	subgenomeB1	DIMADS668.79
Os07t0605200-01	evm.TU.ORIGINAL_1673.404	subgenomeB1	DIMADS1673.404
Os03t0753100-01	evm.TU.FRAGSCAFF_387.1959	subgenomeB1	DIMADS387.1959
Os05t0203800-01	evm.TU.FRAGSCAFF_196.127	subgenomeB1	DIMADS196.127
Os09t0507200-01	evm.TU.ORIGINAL_6123.336	subgenomeB1	DIMADS6123.336
Os12t0207000-01	evm.TU.FRAGSCAFF_194.242	subgenomeB1	DIMADS194.242
Os02t0731200-01	evm.TU.FRAGSCAFF_68.792	subgenomeB1	DIMADS68.792
Os03t0122600-01	evm.TU.FRAGSCAFF_320.519	subgenomeB1	DIMADS320.519
Os02t0170300-01	evm.TU.ORIGINAL_2592.303	subgenomeB1	DIMADS2592.303
Os08t0431900-00	evm.TU.ORIGINAL_609.45	subgenomeB1	DIMADS609.45
Os06t0712700-01	evm.TU.FRAGSCAFF_245.419	subgenomeB1	DIMADS245.419
Os06t0667200-01	evm.TU.FRAGSCAFF_364.12	subgenomeB1	DIMADS364.12
Os08t0112700-01	evm.TU.FRAGSCAFF_40.74	subgenomeB1	DIMADS40.74
Os10t0536100-01	evm.TU.ORIGINAL_2335.34	subgenomeB1	DIMADS2335.34
Os12t0206800-00	evm.TU.FRAGSCAFF_194.243	subgenomeB1	DIMADS194.243
Os02t0761000-01	evm.TU.FRAGSCAFF_68.1016	subgenomeB1	DIMADS68.1016
Os02t0761000-01	evm.TU.FRAGSCAFF_259.345	subgenomeB1	DIMADS259.345
Os06t0347700-00	evm.TU.FRAGSCAFF_392.62	subgenomeB1	DIMADS392.62
Os01t0726400-01	evm.TU.FRAGSCAFF_51.87	subgenomeB1	DIMADS51.87
Os01t0883100-01	evm.TU.FRAGSCAFF_232.107	subgenomeB1	DIMADS232.107
Os01t0883100-01	evm.TU.FRAGSCAFF_343.130	subgenomeB1	DIMADS343.130
Os03t0186600-01	evm.TU.FRAGSCAFF_56.341	subgenomeB1	DIMADS56.341
Os07t0605200-01	evm.TU.FRAGSCAFF_418.287	subgenomeB2	DIMADS418.287
Os03t0753100-01	evm.TU.FRAGSCAFF_387.695	subgenomeB2	DIMADS387.695
Os05t0203800-01	evm.TU.FRAGSCAFF_129.338	subgenomeB2	DIMADS129.338
Os09t0507200-01	evm.TU.ORIGINAL_3556.23	subgenomeB2	DIMADS3556.23
Os12t0207000-01	evm.TU.FRAGSCAFF_351.5	subgenomeB2	DIMADS351.5
Os02t0104100-00	evm.TU.ORIGINAL_1193.29	subgenomeB2	DIMADS1193.29
Os02t0731200-01	evm.TU.FRAGSCAFF_457.237	subgenomeB2	DIMADS457.237
Os03t0122600-01	evm.TU.ORIGINAL_5025.26	subgenomeB2	DIMADS5025.26
Os02t0170300-01	evm.TU.ORIGINAL_6609.29	subgenomeB2	DIMADS6609.29
Os08t0431900-00	evm.TU.ORIGINAL_4580.76	subgenomeB2	DIMADS4580.76
Os06t0712700-01	evm.TU.ORIGINAL_5413.272	subgenomeB2	DIMADS5413.272
Os08t0112700-01	evm.TU.FRAGSCAFF_133.833	subgenomeB2	DIMADS133.833
Os10t0536100-01	evm.TU.FRAGSCAFF_127.336	subgenomeB2	DIMADS127.336
Os02t0761000-01	evm.TU.FRAGSCAFF_156.641	subgenomeB2	DIMADS156.641

Os06t0217300-01	evm.TU.FRAGSCAFF_457.9	subgenomeB2	DIMADS457.9
Os01t0726400-01	evm.TU.ORIGINAL_4442.119	subgenomeB2	DIMADS4442.119
Os01t0883100-01	evm.TU.ORIGINAL_1804.168	subgenomeB2	DIMADS1804.168
Os05t0423400-01	evm.TU.FRAGSCAFF_279.393	subgenomeB2	DIMADS279.393
Os04t0580700-01	evm.TU.FRAGSCAFF_212.288	subgenomeC1	DIMADS212.288
Os02t0682200-01	evm.TU.FRAGSCAFF_244.31	subgenomeC1	DIMADS244.31
Os07t0605200-01	evm.TU.ORIGINAL_3068.29	subgenomeC1	DIMADS3068.29
Os03t0753100-01	evm.TU.FRAGSCAFF_400.119	subgenomeC1	DIMADS400.119
Os03t0215400-01	evm.TU.FRAGSCAFF_218.82	subgenomeC1	DIMADS218.82
Os05t0203800-01	evm.TU.FRAGSCAFF_50.713	subgenomeC1	DIMADS50.713
Os09t0507200-01	evm.TU.ORIGINAL_3472.188	subgenomeC1	DIMADS3472.188
Os12t0207000-01	evm.TU.ORIGINAL_5718.346	subgenomeC1	DIMADS5718.346
Os02t0731200-01	evm.TU.FRAGSCAFF_263.80	subgenomeC1	DIMADS263.80
Os03t0122600-01	evm.TU.FRAGSCAFF_341.158	subgenomeC1	DIMADS341.158
Os02t0170300-01	evm.TU.FRAGSCAFF_199.193	subgenomeC1	DIMADS199.193
Os06t0712700-01	evm.TU.ORIGINAL_4749.49	subgenomeC1	DIMADS4749.49
Os08t0112700-01	evm.TU.FRAGSCAFF_242.44	subgenomeC1	DIMADS242.44
Os10t0536100-01	evm.TU.ORIGINAL_6124.360	subgenomeC1	DIMADS6124.360
Os02t0761000-01	evm.TU.FRAGSCAFF_263.255	subgenomeC1	DIMADS263.255
Os02t0761000-01	evm.TU.FRAGSCAFF_20.313	subgenomeC1	DIMADS20.313
Os01t0726400-01	evm.TU.FRAGSCAFF_173.114	subgenomeC1	DIMADS173.114
Os01t0883100-01	evm.TU.FRAGSCAFF_173.959	subgenomeC1	DIMADS173.959
Os02t0682200-01	evm.TU.FRAGSCAFF_190.946	subgenomeC2	DIMADS190.946
Os02t0579600-00	evm.TU.FRAGSCAFF_445.437	subgenomeC2	DIMADS445.437
Os01t0886200-01	evm.TU.ORIGINAL_4874.41	subgenomeC2	DIMADS4874.41
Os07t0605200-01	evm.TU.ORIGINAL_2114.56	subgenomeC2	DIMADS2114.56
Os03t0753100-01	evm.TU.ORIGINAL_5010.70	subgenomeC2	DIMADS5010.70
Os03t0215400-01	evm.TU.FRAGSCAFF_218.706	subgenomeC2	DIMADS218.706
Os05t0203800-01	evm.TU.ORIGINAL_569.140	subgenomeC2	DIMADS569.140
Os09t0507200-01	evm.TU.FRAGSCAFF_153.269	subgenomeC2	DIMADS153.269
Os12t0207000-01	evm.TU.ORIGINAL_2475.379	subgenomeC2	DIMADS2475.379
Os06t0162800-01	evm.TU.FRAGSCAFF_196.589	subgenomeC2	DIMADS196.589
Os02t0731200-01	evm.TU.FRAGSCAFF_340.1092	subgenomeC2	DIMADS340.1092
Os03t0122600-01	evm.TU.FRAGSCAFF_66.53	subgenomeC2	DIMADS66.53
Os03t0122600-01	evm.TU.ORIGINAL_5125.139	subgenomeC2	DIMADS5125.139
Os02t0170300-01	evm.TU.FRAGSCAFF_175.470	subgenomeC2	DIMADS175.470
Os06t0712700-01	evm.TU.FRAGSCAFF_74.284	subgenomeC2	DIMADS74.284
Os08t0112700-01	evm.TU.FRAGSCAFF_223.1379	subgenomeC2	DIMADS223.1379
Os12t0501700-00	evm.TU.FRAGSCAFF_198.165	subgenomeC2	DIMADS198.165
Os02t0761000-01	evm.TU.FRAGSCAFF_340.1247	subgenomeC2	DIMADS340.1247
Os02t0761000-01	evm.TU.FRAGSCAFF_84.193	subgenomeC2	DIMADS84.193
Os01t0726400-01	evm.TU.FRAGSCAFF_138.122	subgenomeC2	DIMADS138.122
Os01t0883100-01	evm.TU.ORIGINAL_4874.67	subgenomeC2	DIMADS4874.67

Table S4. Summary of the quality of transcriptome sequencing data for floral tissue

Sample	Library	Raw Reads	Clean Reads	Raw Base(G)	Clean Base(G)	Effective Rate(%)	Q30(%)	GC Content(%)
palea	BRRB210005834-1A	21439904	21202528	6.43	6.36	98.89	94.96	52.63
palea1	BRRB210005835-1A	21043999	20746230	6.31	6.22	98.59	94.94	52.65
palea2	BRRB210005836-1A	22875858	22571800	6.86	6.77	98.67	94.65	52.69
pistil	BRRB210005840-1A	23876283	23509875	7.16	7.05	98.47	95.24	52.72
pistil1	BRRB210003611-1A	22616861	22325812	6.79	6.7	98.71	93.03	52.81
pistil2	BRRB210003612-1A	22428694	22259016	6.73	6.68	99.24	93.23	53.24
stamen	BRRB210005841-1A	22344940	22093195	6.7	6.63	98.87	95.04	52.25
stamen1	BRRB210003609-1A	19966031	19745982	5.99	5.92	98.9	92.92	52.77
stamen2	BRRB210003610-1A	21147466	20964211	6.34	6.29	99.13	93.06	53.03

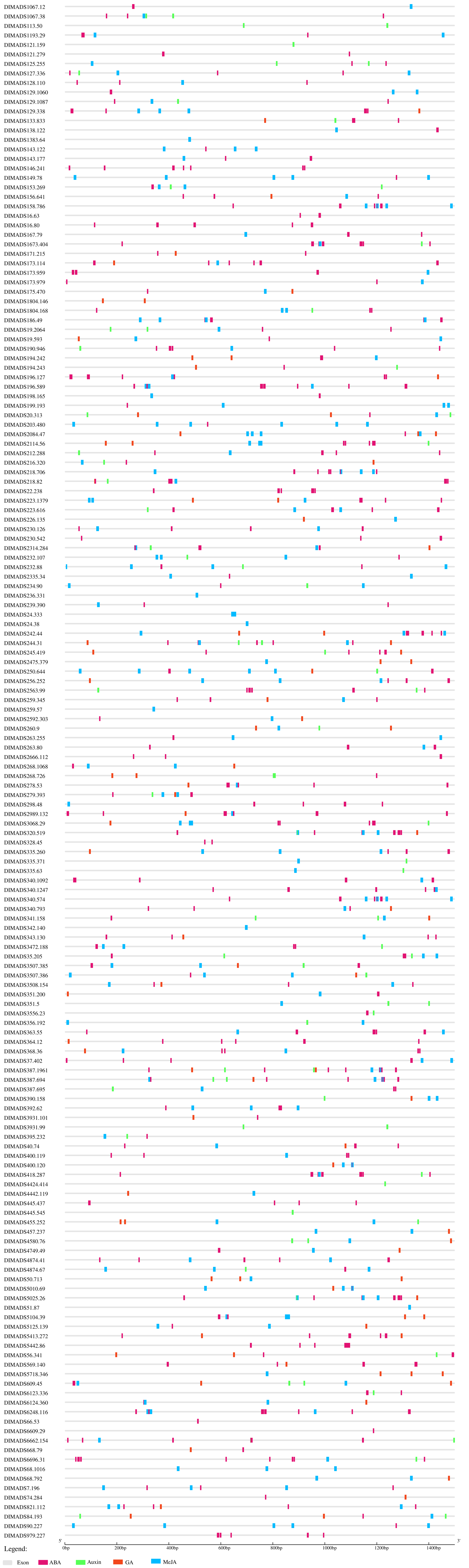

Figure S1 Hormone-associated cis-acting elements in MIKCc -type MADS-box genes

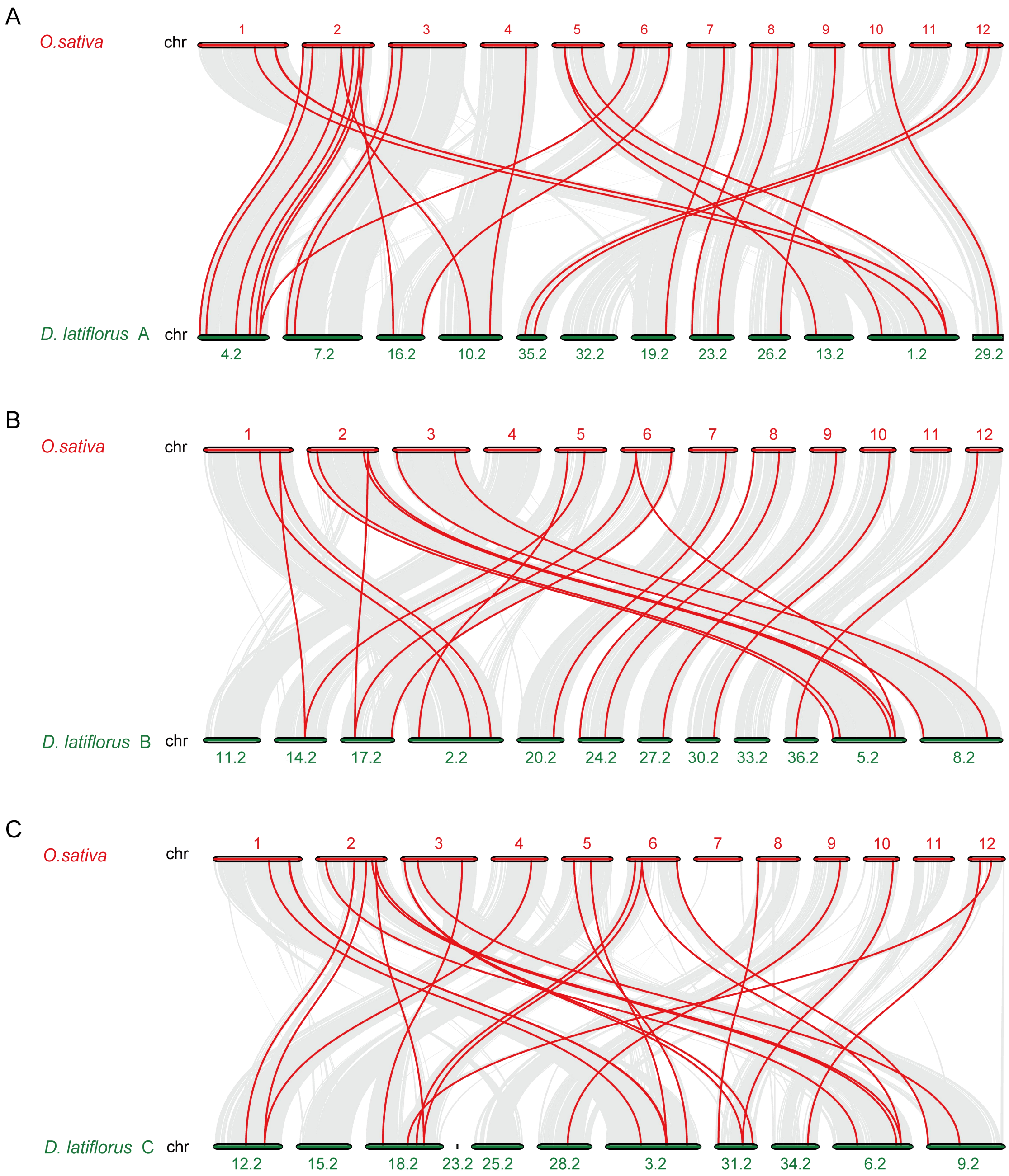

Figure S2. The synteny relationship between D. latiflorus and rice. (A)(B)(C)MIKCc -type MADS-box gene synteny between D. latiflorus subgenome A', B', C' and rice.
